# On the Indications for Instrumental Interference in Labour

**Published:** 1906-10-20

**Authors:** James Morrison

**Affiliations:** Hon. Physician-Accoucheur, Farringdon Dispensary, etc. Being a Demonstration at the Polyclinic


### HOSPITAL CLINICS.

### ON THE INDICATIONS FOR INSTRUMENTAL INTERFERENCE IN LABOUR.

By James Morrison, M.D.(Lond.), Hon. Physician-Accoucheur, Farringdon Dispensary, etc.
Being a Demonstration at the Polyclinic.

The following is a *résumé* of an instructive and practical lecture on the indications for interference in labour cases. The lecturer said:—

The indications for interference during labour depend on general causes in the woman, in the child, and locally in the passages. For example, heart disease may call for urgent interference with the course of labour, whilst the child lying across the pelvis would call for correction as soon as a diagnosis was made.

But in this lecture attention is directed to cases occurring constantly in general practice where there is no gross ailment present, and yet where the use of forceps or turning would greatly benefit the patient and avoid much anxiety and trouble for the practitioner. The call for interference varies greatly with primiparæ and multiparæ, and with the use of the antiseptics of the present day there is rightly a far more frequent use of instruments taking place. Supposing the labour is normal as regards passages and passenger, the average times for the various stages are: In primiparæ, 16 hours for first stage; 8 hours for second stage; half hour for third stage. In multiparæ, 8 hours for first stage; 4 hours for second stage—or, roughly, labour is twice as long in primiparæ as in multiparæ.

The first great rule to be observed is (1) *Never* use forceps if you can possibly help it in primiparæ. The second rule (2) In multiparæ interfere whenever you can safely do so. However expert one may be in the use of forceps, it is a fact that a great deal of damage is done by them, especially so when applied high up; and although the death-rate is not increased, yet the general health and well-being of the patient greatly suffers. Thus quite commonly the vagina is cut or torn on either side of the middle line behind; the cervix lacerated deeply, especially in front (at right angles to the distending force); and the vulva slit under the pubes in front or in varying degrees at the perineum. Now all these tears are dangerous, frequently causing sepsis, but more often causing delay in involution of the pelvic tissues leading to sinking of the parts with prolapse of the uterus and ovaries, retroversions, and, lastly, adhesions between the vaginal walls and pelvis, with subsequent dyspareunia and chronic pains.

The next rule is *never* put on forceps in the first stage—that is to say, before full *dilatation of the os*. This rule is constantly broken in practice, and all sorts of arguments are advanced for doing so. This is an excellent rule, and should be adhered to.

Now what interference is warranted in the first stage in a normal case? Remember as a great axiom that, so long as the membranes are intact, the patient can come to no harm; therefore, however slow the labour is, you are quite safe if you let things alone so long as the membranes are unruptured. In primiparæ never rupture the membranes before the os is two-thirds dilated, and do not do so if the cervix is still thick or the pains weak and irregular. If the cervix is thin, the pains regular and painful, and the os two-thirds dilated, then rupture the membranes and let out the liquor amnii. This will generally shorten labour considerably. Occasionally the cervix may close up again and leave you worse off than before. Only by experience can one learn in which cases to rupture and which to leave alone. In multiparæ, on the other hand, in normal cases it is always justifiable to rupture membranes when the os is the size of a florin, and this will bring on pains which become regular and expulsive.

### Can Anything be done to Correct Prolonged First Stage?

Beyond seeing to the general condition of the patient as regards food and sleep, emptying the bowels and bladder, and giving an occasional hot vaginal douche, it is better not to interfere at all. Never think of giving ergot, and, except possibly quinine, no other drugs will help you. You must wait.

In the first stage delay is most frequently caused (in spite of statistics) by premature rupture of the membranes. The membranes may rupture before labour commences or before the os is fully dilated. The first has been called "premature," and the second "immature" rupture of the membranes, but practically these cases may be classed together. If the membranes are ruptured the woman is no longer safe, but may easily come into danger; and the danger has to be estimated by the condition of the pulse, tongue, and temperature. If the membranes are ruptured before the os is well dilated it is usually, but not always, better to interfere; but if the pulse and temperature are steadily rising, one *must* interfere. In primiparæ (1) if the condition is discovered at the beginning of the first stage, when the head has not begun to descend, the cervix soft, moist, and large enough to admit the finger, the best treatment is to replace the membranes—that is, to insert a *De ribe's bag*, or penny air-ball tied to a catheter. (2) If the head is well in the pelvis, pains weak and irregular, and the cervix dilated to the size of a shilling, soft, moist, and *thick*, again insert the bag, previously pushing up the head. (3) If the head is well down, the cervix thin stretched over head, but os small, with "painful pains," frequent and regular, then do not insert bag, but give chloroform—or chloral per rectum. The same applies if the cervix is rugged, œdematous, and dilated to the size of half a crown. (4) If the temperature is above 100° with dry tongue and rapid pulse, the os still small, the head low in pelvis with caput forming, and the cervix with œdematous anterior lip, then give slight chloroform and dilate with the hand until the cervix can be pushed over head.

In multiparæ premature rupture of membranes is not so important, and, if seen at the commencement of labour, it is justifiable to wait and watch whether the cervix will dilate properly, provided always the case is not an unreduced occipito posterior position, in which case it is always better to insert a De ribe's bag. If descent occurs before the os dilates, the De ribe's bag must also be inserted. It is important to observe that weak pains are never an indication for interference during first stage.

### "Interference in the Second Stage."

The second stage is the period of labour when usually the doctor is able to shorten the labour artificially, and the os being fully dilated and membranes ruptured, it is justifiable to apply forceps or turn.

(1) If the pains are weak and the labour lingers beyond the average time—that is to say, in multiparæ 6 hours, in primiparæ 12 hours (subject to the reservation mentioned before).

(2) If the pulse and temperature are steadily rising and the tongue is becoming furred and dry. In both these cases apply forceps to help the pains, not to replace them.

(3) If the local condition is unsatisfactory the forceps should be applied. If the labour is right the head steadily advances. Failure of this condition is shown by, firstly, the head does not advance; secondly, a caput begins to form, the larger the caput the longer the head has stuck; thirdly, the passages below the head become œdematous; fourthly, the vagina becomes hard, dry, hot—usually a bad case; fifthly, the fœtal heart becomes irregular.

(4) Unfavourable but normal presentations, as for instance in unreduced occipito posterior cases. This is the most frequent case for forceps in general practice.

### Weak Pains.

As regards weak pains it is important to distinguish between those due to general *weakness* and those due to *exhaustion*. The former are present from the commencement, and the uterus at no time has been acting strongly. Locally the presenting part advances, but advances slowly; the caput is not present, and the parts are not œdematous; the vagina is moist, soft, and cool. When due to exhaustion the pains have been strong or violent and have gradually become weaker; the temperature and pulse are raised; the tongue dry; the uterus is tender and irritable; the parts below the presentation have a tendency to be swollen and hard, dry, and hot, but as the pains cease the caput does not increase, and the œdema passes off; the presentation may recede a little, whilst the uterus relaxes and will not respond to stimulation. In the former case the uterus will always contract on irritation, but the contraction is weak and irregular.

### Causes of Delay.

In straightforward cases it is unnecessary to wait so long as four hours in multiparæ after descent has commenced. There are four causes of delay: (1) after the membranes are ruptured the liquor amnii has not escaped, thus preventing the uterus acting directly on the fœtus. (2) The woman is not making proper bearing down with the pains, due probably to improper instruction. This must be regulated. (3) Some alteration in the axis of the genital canal causes the head frequently to stop at the bony outlet. (4) Resistance of the perineum and soft parts—more marked in primiparæ. It should be possible for the patient having good pains to bring the head from the brim to the bony outlet in from two to six good pains. It is commonly at this point that delay occurs, as here the head changes its axis in descent and the uterus acts at a great disadvantage. Pain after pain comes and goes without the head moving, a caput begins to form, and the patient begins to grow exhausted. The proper treatment is to see that the head is properly flexed and that the occiput is in front. Watch the caput and allow half an hour for the head to move. If in a multipara no advance has occurred within that time, in spite of proper regulation of pains and the head being properly flexed, then apply forceps with or without chloroform, and pull the head down into the perineum. In multiparæ it is quite justifiable to keep the blades on until the head is born, but in primiparæ they should always be removed as soon as the perineum is reached in order to allow the perineum to soften. It is practically impossible to save the perineum otherwise; indeed, extensive tearing is common, both of vaginal walls and perineum.

In primiparæ descent from the brim to floor of pelvis usually takes longer than six pains, and so long as the head is descending no caput will form and no interference should be allowed. The parts not having been stretched before are more resistant than in multiparæ, so that the expulsive pains are steadily increasing in order to overcome the resistance. In this way by the time the head reaches the bony outlet the expulsive pains are very urgent and sufficient to push on the head as far as the perineum. Allow the head alternatively to come forwards and retreat as long as possible, at the same time thoroughly foment the perineum to help the parts to relax. If, however, the head becomes *stationary* and a caput begins to form, allow one hour, and then assist with forceps. Take off blades as soon as the head "crowns."

If pulse and temperature are rising, tongue is becoming dry, and patient is becoming tired out, then if there is no contra-indication, apply forceps and help the pains, whether in primiparæ or multiparæ.

It will take some hours for pains to cease entirely from uterine exhaustion, and so long as the uterus is still acting, the patient can safely be delivered; but if the pains have ceased, the danger of post-partum hæmorrhage is enormous, and the woman must be allowed to rest, tr. opii. being given, if necessary, until the pains return, when you can deliver. There is no danger of sloughing, however low the head is, for with the uterus relaxed, the presenting part recedes, and so takes off pressure from the surrounding soft parts, the œdema diminishes and the caput almost disappears. It is only when the uterus is acting strongly and ramming the head into the girdle of contact that delay means danger, sloughing of the parts, etc.

### Tonic Contraction of the Uterus.

If the obstacle to delivery is insurmountable, the uterus, acting at first strongly, rapidly goes into tonic contraction, until at length the pains cease; and in these cases the condition is quite different from that just described. The presenting part is rammed tightly into the passage, the parts below become more and more œdematous, whilst the patient's pulse and temperature rapidly rise and general exhaustion supervenes. In such cases no delay is allowable, but the patient is to be delivered at once. This condition is rarely seen apart from obstructed labour. In multiparæ with weak pains, but normal passage and passenger, when the head will not advance and it is justifiable to help the patient, forceps are usually selected; but many cases can be more easily delivered by turning. The patient has a dread of instruments, whereas the hand can be inserted without frightening her. Turning may be performed so long as the uterus is relaxed in between the pains, and the head can be pushed back out of the pelvis; but remember in turning always push the presenting part up again above the brim before attempting to seize the leg.

### Potassium Iodide in Aneurysm.

Mr. Eric E. Young describes an example of the value of full doses of potassium iodide in aneurysm. The sac protruded so considerably that fear of external rupture led to the daily application of collodion. Yet after some weeks pulsation disappeared from the swelling under the influence of rest, restricted diet, and potassium iodide, the latter in gradually increasing doses up to a maximum of 60 grains thrice daily.

